# Identification of PCB Congeners and their Thresholds associated with Diabetes using Decision Tree Analysis

**DOI:** 10.21203/rs.3.rs-2845995/v1

**Published:** 2023-05-01

**Authors:** Tuo Lan, Buyun Liu, Wei Bao, Peter S. Thorne

**Affiliations:** University of Iowa College of Public Health; University of Science and Technology of China; University of Science and Technology of China; University of Iowa

## Abstract

Few studies have investigated the potential combined effects of multiple PCB congeners on diabetes. To address this gap, we used data from 1244 adults in the National Health and Nutrition Examination Survey (NHANES) 2003–2004. We used 1) classification trees to identify serum PCB congeners and their thresholds associated with diabetes; and 2) logistic regression to estimate the odds ratios (ORs) and 95% confidence intervals (CIs) of diabetes with combined PCB congeners. Of the 40 PCB congeners examined, PCB 126 has the strongest association with diabetes. The adjusted OR of diabetes comparing PCB 126 > 0.025 to ≤ 0.025 ng/g was 2.14 (95% CI 1.30–3.53). In the subpopulation with PCB 126 > 0.025 ng/g, a lower PCB 101 concentration was associated with an increased risk of diabetes (comparing PCB 101 < 0.72 to ≥ 0.72 ng/g, OR = 3.3, 95% CI: 1.27–8.55). In the subpopulation with PCB 126 > 0.025&PCB 101 < 0.72 ng/g, a higher PCB 49 concentration was associated with an increased risk of diabetes (comparing PCB 49 > 0.65 to ≤ 0.65 ng/g, OR = 2.79, 95% CI: 1.06–7.35). This nationally representative study provided new insights into the combined associations of PCBs with diabetes.

## Introduction

An estimated 537 million people’s lives had been affected by diabetes in 2019 worldwide, and this number is estimated to rise up to 643 million by 2030, making diabetes a growing epidemic^1^. Although risk factors such as weight and physical activity have been identified, there has been increasing evidence suggesting that exposure to environmental chemicals can also be important for diabetes development^2,3^.

Polychlorinated biphenyls (PCBs), a group of persistent and carcinogenic chemicals, are still being produced inadvertently after the ban in 1978^4,5^. PCBs have been suspected to contribute to diabetes risks by acting as endocrine disruptors^6–9^. Epidemiological studies have found that higher serum concentrations of PCBs were associated with an increased risk of diabetes in different cohorts across the world^10–17^. This is supported by biologically plausible molecular mechanisms including altering gene transcription and lipid metabolism, changes in insulin production and signaling pathway, adipose inflammation, and impairment of glucose homeostasis^18,19^. Even though PCBs are a mixture of 209 congeners with district biophysicochemical properties, previous research has focused on individual PCB congeners (e.g., PCB 126, PCB 138, PCB 153, and PCB 155) or specific PCB metrics (e.g., dioxin-like and non-dioxin-like, low- and high-chlorination PCBs) in serum^10–17^, with no study on potential combined effects of the different PCB congeners on diabetes. In fact, multipollutant analyses are important and receiving growing attention because of the potential additive, synergistic or antagonistic effects among the chemicals^20–22^.

To advance our understanding of the role of serum PCBs in diabetes, we used nationally representative data from the National Health and Nutrition Examination Survey (NHANES) to: 1) identify PCB congeners and their thresholds that could be associated with diabetes; and 2) examine the association of the identified PCB congener profiles and their combined associations with diabetes.

## Methods

### Study Design and Population

The NHANES is an ongoing study conducted by the National Center for Health Statistics (NCHS) of the Centers for Disease Control and Prevention (CDC). The study uses a complex, multistage, probability sampling strategy to include an over-sampling of minorities and to represent national non-institutionalized U.S. populations^23^. Information on sociodemographic characteristics, lifestyle characteristics, diet, and medical conditions are collected via an in-person interview and a physical examination in a mobile examination center (MEC), respectively. The NHANES data are released publicly every two years. The study was approved by the National Center for Health Statistics (NCHS) Research Ethics Review Board.

For this study, we used data from NHANES 2003–2004 because it provided the most recent measurements of serum PCBs for each participant. We limited the analysis to non-pregnant adults aged ≥ 20 years who had data available on serum PCBs and diabetes information (n = 1,258). Additional exclusions were individuals whose body mass index (BMI) data were unavailable (n = 30) and individuals with missing covariate information (n = 4). As a result, 1,224 adult participants were included in the study.

### Exposure Assessment

Serum PCBs were measured by high-resolution gas chromatography-mass spectrometry (HRGC/ID-HRMS) among a randomly selected one-third of participants who were 12 years old or older. Briefly, around 2–10 ml of serum sample spiked with 13C-labeled internal standards were extracted using a C18 solid phase extraction (SPE) procedure with hexane^24^. Each congener had a specific limit of detection. According to NHANES analytic guidance, values below LOD were assigned the value of LOD divided by the square root of 2.

A total of 40 PCB congeners were quantified, they were PCB 28, 44, 49, 52, 66, 74, 81, 87, 99, 101, 105, 110, 118, 126, 128, 138 + 158, 146, 149, 151, 153, 156, 157, 167, 169, 170, 172, 177, 178, 180, 183, 187, 189, 194, 195, 196 + 203, 199, 206, and 209. Because PCB 138 coeluted with PCB 158 and PCB 196 coeluted with PCB 203, the 40 PCB congeners were included in the analyses as 38 variables. Serum PCB concentrations were included in lipid adjusted forms because they are lipophilic.

### Diabetes Ascertainment

Diabetes status was ascertained through a self-reported questionnaire by trained interviewers and lab tests. Specifically, participants were defined as having diabetes if they reported having been previously diagnosed with diabetes by a physician, or undiagnosed diabetes but had glycohemoglobin (A1C) ≥ 6.5% or plasma fasting glucose concentrations ≥ 126 mg/dL^25,26^. This method of diabetes ascertainment was found to be 63.2% sensitive and 97.4% specific for diabetes in a previous NHANES validation study^27^.

### Sociodemographic and Lifestyle Characteristics Assessment

Information on age, sex (male/female), race/ethnicity (non-Hispanic White, non-Hispanic Black, Hispanic, and other), education (less than high school, high school, and higher than high school), family history of diabetes (yes/no), family income, smoking status, alcohol consumption, and physical activity was assessed by self-reported questionnaires during the in-person interview. Family income-to-poverty ratio (PIR) was categorized as ≤ 1.30, 1.31–3.50, and > 3.50^28^. Smoking status was categorized as never (smoked less than 100 cigarettes in their lifetime), ever (not smoke at the time of the survey) and current smoker (smoke at the time of the survey)^29^ Physical activity was categorized as < 600, 600–1200, and > 1200 metabolic equivalents of task (MET) min per week^30^. Weight and height were measured following a standardized protocol during the physical examination, and BMI was calculated as weight in kilograms divided by height in meters squared. BMI categories were defined as underweight (< 18.5 kg/m^2^), normal (18.5–24.9 kg/m^2^), overweight (25.0–29.9 kg/m^2^), and obese (≥ 30.0 kg/m^2^). Sixteen underweight participants were combined with normal-weight participants for statistical analyses. Dietary information was obtained through 24-h dietary recall. Total energy intake (kcal/day) and alcohol intake were calculated using the USDA food composition database. Alcohol intake was then categorized as non-drinker (0 g/day), moderate drinker (0.1–28 g/day for men and 0.1–14 g/day for women), and heavy drinker (≥ 28 g/day for men and ≥ 14 g/day for women)^31^. Diet quality, represented by Healthy Eating Index − 2010 (HEI), has been found to be associated with a decreased risk of diabetes^32^. A higher HEI score indicates a higher diet quality based on 12 food components including total fruit, whole fruit, total vegetables, greens and beans, whole grains, dairy, total protein foods, seafood and plant proteins, fatty acids, refined grains, sodium, and empty calories (e.g., added sugars)^31^.

### Statistical Analysis

For descriptive statistical analyses, we accounted for the complex, multistage design of NHANES by using appropriate sample weights, strata, and primary sampling units. We compared population characteristics by quintile of lipid adjusted serum concentration of the sum of 40 PCBs (∑40-PCBs) using the t-test for continuous variables and the chi-square test for categorical variables. Then, we examined the potential combined effects of the 40 PCB congeners on diabetes in two steps.

In our first step, we used the decision tree classification model to identify serum PCB profiles in relation to diabetes with a corresponding threshold. The classification tree, a non-parametric supervised learning method, was chosen for several reasons. First, it can perform dimensionality reduction and classification simultaneously, which is helpful for analyzing serum PCBs, a complex mixture of different congeners. Second, it can identify potential interactions among a mixture of PCBs. Third, it can identify threshold values for each PCB congener. Last, it is robust for outliers of PCBs and does not have to make assumptions about data distributions. The participants were classified as living with diabetes or not based on all measured 40 PCB congeners. The entire dataset was randomly split into 70% training sets (n = 858) and 30% test sets (n = 386). And a ten-fold cross-validation procedure was used to optimize the parameters and prune the tree to avoid overfitting. We used the confusion matrix and computed the accuracy with test sets to evaluate the tree’s performance. This analysis was performed using the rpart package in R version 4.1.2.

In our second step, logistic regression was used to estimate odds ratios (ORs) and 95% confidence intervals (CIs) of diabetes associated with the identified serum PCB profiles. We followed NHANES analytic guidelines accounting for sample weights and sample design. In the basic models, we adjusted for only demographic variables including age, gender and race/ethnicity. In the full models, we additionally adjusted for variables that could serve as potential confounders including BMI, education level, family income to poverty ratio, smoking status, alcohol intake, physical activity level, 2010 healthy eating index, and family history of diabetes.

Although NHANES does not explicitly collect information on the type of diabetes, we considered participants to have type 1 diabetes if they started insulin within one year of diabetes diagnosis, or were currently using insulin, or were diagnosed with diabetes under age 30 [62]. To explore the influence of diabetes type, we performed a sensitivity analysis excluding those possible type 1 diabetes cases; therefore, the vast majority of the remaining cases would be type 2 diabetes cases. This second step was performed using survey procedures with SAS software (version 9.4; SAS Institute Inc., Cary, NC, USA).

## Results

Among the 1,224 eligible participants, their weighted mean (SE) age was 46 (0.6) years old, 50.8% (95% CI = 47.2%–54.4%) were female and 70.9% (95% CI = 64.0%–77.7%) were non-Hispanic White. The prevalence of diabetes was 13.2% in the study population and the weighted median of serum concentration of the sum of 40 PCBs (∑40-PCBs) was 153.9 ng/g lipid adjusted (interquartile range [IQR] 87.9–266.4). Compared to participants with a lower serum concentration of ∑40-PCBs, those with a higher serum concentration of ∑40-PCBs were more likely to be older, have a lower total energy intake, a better dietary quality as assessed by the HEI-2010, and diabetes; and less likely to be Hispanic, current smokers, and have a lower family income (Table 1).

**Figure 1:**
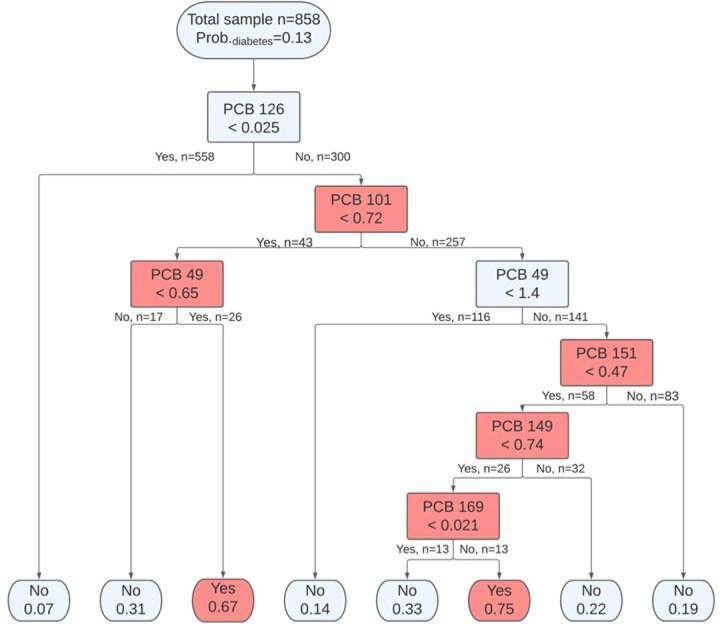
Classification tree from step one analysis of serum PCB congeners (ng/g lipid adjusted weight) and diabetes status, NHANES 2003–2004. The root node and internal nodes present the identified PCB profiles including PCB congeners and their thresholds; the leaf nodes (endpoints) present the predicted diabetes status (No: non-diabetes and Yes: diabetes) with its probability. The red color indicates a higher probability of having diabetes.

Using a non-parametric supervised learning method, a classification tree consisting of a combination of PCB congeners and their thresholds that related to diabetes were learned among the 858 training samples (Fig. 1). Identified PCB profiles that related to diabetes were indicated in the internal nodes. Each node separated the participants into two more homogeneous subpopulations based on whether their serum PCB concentrations were higher or lower than the threshold. The probability of having diabetes in the subpopulation and the proportion of subpopulation were indicated above each identified PCB profile. At the root node, the PCB profile (ng/g lipid weight) most related to diabetes was identified: participants with serum concentration of PCB 126 ≥ 0.025 had a higher probability of having diabetes (probability = 0.24). Among participants with serum concentration of PCB 126 ≥ 0.025, additional six PCB profiles with PCB 101, 49, 151, 149, and 169 were also identified by the tree for predicting diabetes. They were PCB 126 > 0.025 & PCB 101 < 0.72 (probability = 0.43), PCB 126 > 0.025 & PCB 101 < 0.72 & PCB 49 ≥ 0.65 (probability = 0.67), PCB 126 > 0.025 & PCB 101 ≥ 0.72 & PCB 49 ≥ 1.4 (probability = 0.27); PCB 126 > 0.025 & PCB 101 ≥ 0.72 & PCB 49 ≥ 1.4& PCB 151 < 0.47 (probability = 0.40), PCB 126 > 0.025 & PCB 101 ≥ 0.72 & PCB 49 ≥ 1.4 & PCB 151 < 0.47 & PCB 149 ≥ 0.74 (probability = 0.55), and PCB 126 > 0.025 & PCB 101 ≥ 0.72 & PCB 49 ≥ 1.4 & PCB 151 < 0.47 & PCB 149 ≥ 0.74 & PCB 169 ≥ 0.021 (probability = 0.75). The accuracy rate of the model on test data was 0.842, which indicates the model could predict 84.2% of the samples correctly.

Table 2 presents adjusted ORs and 95% CI of diabetes risk by the identified PCB profiles. After adjusting for confounders, PCB 126 was still the most consistent congener associated with diabetes; the ORs (95% CIs) of diabetes were 2.11 (1.24–3.61) in the basic model and 2.14 (1.30–3.53) in the full model for participants with a higher serum concentration of PCB 126 (> 0.025 ng/g), compared to those with a lower PCB 126 (≥ 0.025 ng/g). Interestingly, in the subpopulation with a higher serum concentration of PCB 126, a lower serum concentration of PCB 101 was associated with an increased risk of diabetes (comparing PCB 101 < 0.72 to ≥ 0.72 ng/g, fully adjusted OR = 3.3, 95% CI: 1.27–8.55). In the subpopulation with a higher serum concentration of PCB 126 and a lower serum concentration of PCB 101, a higher serum concentration of PCB 49 was associated with an increased risk of diabetes (comparing PCB 49 > 0.65 to ≤ 0.65 ng/g, fully adjusted OR = 2.79, 95% CI: 1.06–7.35). Although the last two identified PCB profiles with PCB 126, 101, 49, 151, 149, and 169 were also significantly associated with diabetes, these findings were inconclusive because of the wide confidence intervals. In the sensitivity analyses excluding those who possibly had type 1 diabetes, similar results were observed (Supplemental table 1).

## Discussion

In this nationally representative sample of US adults, we identified serum PCB congeners and their thresholds on diabetes using classification tree analysis. After adjustment for demographic, socioeconomic, dietary, and lifestyle factors, we found that serum PCB 126 was the congener that was most consistently associated with diabetes. Further, we identified the combined associations of serum PCB 126, 101, and 46 with diabetes.

Our finding that a higher serum concentration of PCB 126 was associated with an increased risk of diabetes in the NHANES 2003–2004 was consistent with the previous findings in the NHANES 1999–2002 and in a Belgian study^11,17^. Comparing our threshold of PCB 126 identified by classification tree to that in the NHANES 1999–2002, our threshold (≥ 0.025 ng/g) were lower than their medium group (0.031–0.084 ng/g) and high group (≥ 0.084 ng/g) that associated with total diabetes (medium vs. low OR = 1.67, 95% CI: 1.03–2.71 and high vs. low OR = 3.68, 95% CI: 2.09–6.49). PCB 126 was the most consistent congener associated with diabetes is plausible because it is the most potent dioxin-like PCB congener that can interact with the aryl hydrocarbon receptor (AhR), alter glucose transport and insulin tolerance in mice through an AhR-dependent mechanism^33–35^, and inhibit adipogenesis which leads to alteration in fatty acid metabolism^36^.

With respect to the findings of the combined associations, to our best knowledge, the only other comparable study is a recently published study that compared the multipollutant effects of persistent organic pollutants (POPs) mixture exposure on gestational diabetes mellitus (GDM) risk^37^. That study evaluated six non-dioxin-like (DNL) PCBs (PCB 28, 52, 101, 138, 153, and 180) with other POPs and found that PCB 101 was the most important predictor for glucose homeostasis but the least important predictor for GDM. This discrepancy and our finding that PCB 101 was negatively associated with diabetes among participants with a higher PCB 126 can be explained by several possible mechanisms including PCB metabolism and interaction between PCB mixtures and diabetes. Because PCB 101 can be rapidly metabolized through cytochrome P450 (CYP) 3A family enzymes, and PCB 126 can induce activation of CYP 3A^38,39^, PCB 126 may accelerate the metabolism of PCB 101. As the Liu et al. study did not include PCB 126 in the analysis, it is possible that the observed positive association between PCB101 and GDM actually reflects the effect of PCB126. In addition, it is very common that environmental exposure and health outcomes are not linearly associated. The non-linear relationship between PCB 101 and GDM was observed among pregnant women in a prior study^40^. Inverse associations of GDM with PCB 101 at relatively low or high concentrations were shown in their dose-response curves. Although GDM tends to be a temporary condition, the risk of developing diabetes is 10-fold higher among women with GDM history than those with no GDM history^41^. In the subpopulation with a higher PCB 126 and a lower PCB 101, our observed positive association between PCB49 and diabetes was expected. This might be explained by the estrogenic activity of PCB 49 that can disrupt normal endocrine function^42^. However, this finding was different from those in an Anniston cohort study that observed a null association between estrogenic congener group (PCB 44, 49, 66, 74, 99, 110, and 128) and diabetes^16^. The difference in PCBs examined (specific PCB profile vs. the sum of 7 estrogenic congeners), race/ethnicity (national representative vs. 46% African American), exposure level (general population vs. highly exposed) likely complicated the comparison of the findings.

A major strength of this analysis was that we used data-driven approach to analyze a complex mixture of serum PCBs, which can assess the associations between 40 serum PCB congeners and diabetes simultaneously. Another strength was the use of nationally representative data from NHANES, which allows us to generalize our findings to the population of the U.S. This study also had some limitations. First, we examined the combined associations of PCBs with diabetes in a smaller subpopulation with a higher serum PCB 126 concentration. Although this method can provide interpretable results for the exposed populations, the referent groups were different populations of varying size. Thus, we cannot compare the magnitude of the observed associations across the subpopulation. Second, we cannot establish a temporal relation for the observed association between PCBs and diabetes because of the cross-sectional study design. Third, as the NHANES study does not differentiate type 1 from type 2 diabetes, we cannot definitively distinguish the effects on type 1 and type 2 diabetes separately. Since type 2 diabetes contributes 90% or more of total diabetes in adults in the U.S.^43^, the observed association was likely to be largely reflected by type 2 diabetes. In addition, we performed a stratified analysis excluding those who possibly had type 1 diabetes, and found similar findings as in our main analysis. Finally, although we controlled a variety of confounders, the potential for residual confounding could remain.

## Conclusions

In conclusion, in one of the few studies to investigate the combined associations of PCBs with diabetes risk, we identified serum PCB congeners and their thresholds associated with diabetes using classification tree analysis. Our findings provide new insights into the combined associations of PCBs with diabetes. Additional prospective studies with more detailed diabetes type information are needed to replicate these findings.

## Figures and Tables

**Table 1:** Population characteristics by quintiles of total serum PCB concentrations in NHANES 2003–2004

	Σ40 PCBs (ng/g lipid weight)					
	Q1 (< 77)	Q2 (77–142)	Q3 (142–230)	Q4 (230–361)	Q5 (≥ 361)	P-value
Number of Participants	244	245	245	245	245	
Age, years	30.0 (0.7)	37.5 (0.8)	48.0 (1.0)	58.3 (1.0)	64.9 (1.2)	**<0.001**
Gender						0.445
Male	45.9 (1.2)	54.0 (1.3)	47.6 (1.0)	46.4 (0.9)	51.4 (0.9)	
Female	54.1 (1.1)	46.60(1.2)	52.44(1.2)	53.6 (0.9)	48.6 (0.7)	
Race/ethnicity						**<0.001**
Hispanic	26.9 (0.9)	9.8 (0.6)	10.4 (0.7)	4.7 (0.3)	6.6 (0.4)	
Non-Hispanic white	57.0 (1.2)	73.4 (1.5)	73.1 (1.5)	79.6 (1.6)	72.1 (0.7)	
Non-Hispanic black	10.2 (0.6)	9.1 (0.6)	9.1 (0.4)	10.4 (0.5)	15.4 (0.7)	
Other	5.9 (0.5)	7.8 (0.7)	7.4 (0.6)	5.3 (0.4)	5.9 (0.3)	
Education						**0.015**
Less than high school	21.5 (0.7)	16.3 (0.7)	12.2 (0.6)	21.3 (0.7)	24.2 (0.7)	
High school	27.1 (0.9)	20.5 (0.6)	23.4 (0.8)	30.0 (0.6)	26.1 (0.5)	
More than high school	51.4 (0.8)	63.1 (1.6)	64.3 (1.1)	48.7 (0.9)	49.7 (1.1)	
Family income to poverty ratio						**0.034**
<1.3	24.8 (0.9)	20.6 (0.7)	19.2 (0.6)	14.6 (0.6)	14.3 (0.4)	
1.3–3.5	39.4 (1.1)	34.5 (1.2)	34.3 (1.0)	37.9 (0.5)	40.7 (0.4)	
>3.5	29.2 (1.1)	42.2 (1.2)	44.3 (1.0)	40.4 (1.0)	39.3 (0.9)	
Smoking						**<0.001**
Never	55.4 (0.8)	52.5 (1.2)	46.4 (1.0)	45.5 (1.1)	50.5 (1.0)	
Ever	14.0 (0.3)	19.9 (0.7)	27.7 (0.7)	34.7 (1.0)	31.8 (0.8)	
Current	30.6 (0.8)	27.6 (0.9)	25.8 (1.0)	19.8 (0.6)	17.7 (0.4)	
Alcohol						0.989
Non	69.4 (1.0)	67.7 (1.3)	71.7 (1.5)	71.2 (1.2)	66.8 (0.7)	
Moderate	6.1 (0.5)	8.1 (0.5)	5.3 (0.4)	6.6 (0.4)	7.3 (0.3)	
Heavy	19.1 (0.7)	19.2 (0.8)	17.1 (0.7)	18.5 (0.6)	19.0 (0.9)	
BMI						**0.004**
Normal/underweight	30.4 (0.6)	46.1 (1.6)	37.5 (0.9)	24.7 (0.8)	38.2 (0.8)	
Overweight	33.7 (0.8)	30.2 (0.9)	30.1 (1.2)	34.1 (0.7)	36.2 (0.8)	
Obese	35.9 (0.9)	23.7 (0.8)	32.5 (0.9)	41.3 (0.9)	25.6 (0.6)	
Physical Activity, MET-min/week						0.262
<600	43.4 (0.9)	39.9 (1.2)	39.7 (1.4)	38.7 (0.9)	46.4 (0.7)	
600–1199	14.4 (0.4)	11.9 (0.5)	20.0 (0.8)	20.8 (0.5)	16.3 (0.4)	
≥1200	42.2 (1.2)	48.1 (1.5)	40.3 (1.1)	40.6 (0.6)	37.2 (0.7)	
Total energy intake (kcal/day)	2553 (79)	2394 (81)	2237 (86)	2138 (83)	1942 (95)	**0.006**
HEI-2010	45.6 (1.2)	47.2 (1.2)	48.8 (1.1)	48.5 (1.0)	51.1(0.9)	**<0.001**
Diabetes						**<0.001**
No	3.5 (0.3)	5.1 (0.4)	10.2 (0.8)	11.6 (0.5)	26.5(0.6)	
Yes	96.5 (1.4)	94.9 (1.9)	89.8 (1.1)	88.4 (1.1)	73.5(1.1)	

Data are presented as the weighted mean and standard error for continuous variables; and weighted percentages and standard error for categorical variables. Some percentages may not sum to 100% because of missing values.

BMI, body mass index; HEI-2010, 2010 healthy eating index; MET; metabolic equivalent of task.

**Table 2:** Multivariable-adjusted odd ratios (ORs) and 95% confidence intervals (CIs) of diabetes by the combined associations of PCB congeners, NHANES 2003–2004.

PCB profiles (ng/g lipid weight)	No. of exposure/No. of subgroup population	Reference	ORs (95% CI)
PCB 126 > 0.025	416/1224	PCB126 ≤ 0.025	
Basic model^1^		1	**2.11 (1.24–3.61)**
Fully adjusted model^2^		1	**2.14 (1.30–3.53)**
PCB 126 > 0.025 & PCB 101 < 0.72	68/416	PCB126 > 0.025 & PCB 101 ≥ 0.72	
Basic model^1^		1	**2.61 (1.05–6.47)**
Fully adjusted model^2^		1	**3.30 (1.27–8.55)**
PCB 126 > 0.025 & PCB 101 < 0.72 & PCB 49 ≥ 0.65	29/68	PCB126 > 0.025 & PCB 101 < 0.72 & PCB 49 < 0.65	
Basic model^1^		1	**4.21 (1.65–10.8)**
Fully adjusted model^2^		1	**2.79 (1.06–7.35)**
PCB 126 > 0.025 & PCB 101 ≥ 0.72 & PCB 49 ≥ 1.4	193/348	PCB126 > 0.025 & PCB 101 ≥ 0.72 & PCB 49 < 1.4	
Basic model^1^		1	1.29 (0.54–3.13)
Fully adjusted model^2^		1	2.03 (0.71–5.81)
PCB 126 > 0.025 & PCB 101 ≥ 0.72 & PCB 49 ≥ 1.4 & PCB 1 51 < 0.47	79/223	PCB 126 > 0.025 & PCB 101 ≥ 0.72 & PCB 49 ≥ 1.4 & PCB 151 ≥ 0.47	
Basic model^1^		1	**3.09 (1.02–9.34)**
Fully adjusted model^2^		1	2.31 (0.78–6.82)
PCB 126 > 0.025 & PCB 101 ≥ 0.72 & PCB 49 ≥ 1.4 & PCB 1 51 < 0.47 & PCB 149 ≥ 0.74	40/79	PCB126 > 0.025 & PCB 101 ≥ 0.72 & PCB 49 ≥ 1.4 & PCB 151 < 0.47 & PCB 49 < 0.74	
Basic model^1^		1	3.96 (0.95–16.5)
Fully adjusted model^2^		1	**13.5 (2.21–82.5)**
PCB 126 > 0.025 & PCB 101 ≥ 0.72 & PCB 49 ≥ 1.4 & PCB 1 51 < 0.47 & PCB 149 ≥ 0.74 & PCB 169 ≥ 0.021	19/40	PCB 126 > 0.025 & PCB 101 ≥ 0.72 & PCB 49 ≥ 1.4 & PCB 151 < 0.47 & PCB 49 ≥ 0.74 &PCB 169 < 0.021	
Basic model^1^		1	**11.4 (2.11–61.6)**
Fully adjusted model^2^		1	**Very large or infinite** ^3^

1 Basic model was adjusted for age, sex, race/ethnicity

2 Full model was adjusted for age, sex, race/ethnicity, BMI, education level, Family income to poverty ratio, smoking status, alcohol intake, physical activity level, 2010 healthy eating index, and family history of diabetes.

3 The fully adjusted odd ratio was very large due to the small sample size. Some covariates had few observations in the sub-category group (e.g., only three people had diabetes were normal weight).

## Data Availability

The data are publicly available at NHANES’s website.
